# *N*,*N*-dimethylformamide induces cellulase production in the filamentous fungus *Trichoderma reesei*

**DOI:** 10.1186/s13068-019-1375-1

**Published:** 2019-02-19

**Authors:** Yumeng Chen, Chuan Wu, Yaling Shen, Yushu Ma, Dongzhi Wei, Wei Wang

**Affiliations:** State Key Lab of Bioreactor Engineering, East China University of Science and Technology, 130 Meilong Road, P.O.B. 311, Shanghai, 200237 China

**Keywords:** *Trichoderma reesei*, DMF induced, Cellulase, Calcium signaling, Permeability, Phospholipase C, *plc*-*e*

## Abstract

**Background:**

The filamentous fungus *Trichoderma reesei* produces cellulase enzymes that are widely studied for lignocellulose bioconversion to biofuel. *N*,*N*-dimethylformamide (DMF) is a versatile organic solvent used in large quantities in industries.

**Results:**

In this study, we serendipitously found that biologically relevant concentrations of extracellular DMF-induced cellulase production in the *T. reesei* hyper-cellulolytic mutant Rut-C30 and wild-type strain QM6a. Next, by transcriptome analysis, we determined that *plc*-*e* encoding phospholipase C was activated by DMF and revealed that cytosolic Ca^2+^ plays a vital role in the response of *T. reesei* to DMF. Using EGTA (a putative extracellular Ca^2+^ chelator) and LaCl_3_ (a plasma membrane Ca^2+^ channel blocker), we demonstrated that DMF induced a cytosolic Ca^2+^ burst via extracellular Ca^2+^ and Ca^2+^ channels in *T. reesei*, and that the cytosolic Ca^2+^ burst induced by DMF-mediated overexpression of cellulase through calcium signaling. Deletion of *crz1* confirmed that calcium signaling plays a dominant role in DMF-induced cellulase production. Additionally, 0.5–2% DMF increases the permeability of *T. reesei* mycelia for cellulase release. Simultaneous supplementation with 1% DMF and 10 mM Mn^2+^ to *T. reesei* Rut-C30 increased cellulase activity approximately fourfold compared to that without treatment and was also more than that observed in response to either treatment alone.

**Conclusions:**

Our results reveal that DMF-induced cellulase production via calcium signaling and permeabilization. Our results also provide insight into the role of calcium signaling in enzyme production for enhanced cellulase production and the development of novel inducers of cellulase.

**Electronic supplementary material:**

The online version of this article (10.1186/s13068-019-1375-1) contains supplementary material, which is available to authorized users.

## Background

Lignocellulose, the most abundant renewable resource, has attracted great attention for its ability to hydrolyze sugars for the production of biofuels and chemicals [1, 2]. Deconstruction of lignocellulose in an economical way, however, is critical to the economic viability of cellulase production [3, 4]. As one of the most prominent cellulase and hemicellulase producers in nature, the filamentous fungus *Trichoderma reesei* has been widely studied for the production of commercial cellulase [5, 6].

The most abundantly secreted and industrially interesting cellulases derived from *T. reesei* are the two main cellobiohydrolases, CBHI and CBHII (EC 3.2.1.91), and two of the major endo-β-1,4-endoglucanases, EGI and EGII (EC 3.2.1.4) [7]. The majority of the cellulase genes are regulated by transcription factors (TFs). Of these, XYR1 is the main transcriptional activator of cellulase genes in *T. reesei* [8]. Additionally, the full expression of high-level cellulase for industrial purposes requires the presence of inducers, mainly including cellulose, cellobiose, lactose, and sophorose [6, 9, 10], which have long been recognized. Finding novel inducer exhibiting high efficiency and low cost is always a priority for investigators [11]. Additionally, a wide diversity of induction signals, including calcium signaling, are present during cellulase production and are able to regulate the expression of cellulase-encoding genes [12, 13]. The sensing signals for induction and regulation of cellulase in *T. reesei*, however, remain largely unknown.

Cellular Ca^2+^, a ubiquitous second messenger, is an important intracellular signaling molecule involved in regulating primary and secondary metabolism in microorganisms such as fungi [14]. Ca^2+^ is also the key component of the calcium signal transduction pathway in all fungi [15, 16]. Liu et al. [17] demonstrated that calcium activates calmodulin to regulate ganoderic acid biosynthesis in *Ganoderma lucidum*, a well-known medicinal basidiomycete, and Chen et al. [13] revealed that calcium signaling pathways participate in cellulase production in *T. reesei*. In our previous study, we demonstrated that Mn^2+^ induction of calcium signaling participates in regulating cellulase gene expression in *T. reesei* [18]. Under specific conditions, including exposure to high temperatures or high concentrations of Mn^2+^, cytosolic Ca^2+^ concentrations can be increased in microorganisms to transmit downstream signals that regulate various cellular processes [18–22]. Cytosolic Ca^2+^ bursts activate downstream components of the pathway, such as calmodulin (Cam), calcineurin (Cna), and calcineurin-responsive zinc finger transcription factor 1 (Crz1 or CrzA), and these then participate in secondary metabolism in response to extracellular signals. An increase in cytosolic Ca^2+^ levels leads to activation of calcineurin by Ca^2+^/calmodulin, where activated calcineurin dephosphorylates Crz1/CrzA, and then activated Crz1/CrzA induces transcription of downstream genes [23]. Chen et al. [13] verified that CRZ1 (a Crz1/CrzA homolog in *Trichoderma*) can bind directly to upstream regions of the cellulase gene *cbh1*. Therefore, increasing cytosolic Ca^2+^ concentrations in *T. reesei* is important for cellulase overexpression.

An increase in cytosolic Ca^2+^ may affect two pathways. First, extracellular Ca^2+^ can enter the cytoplasm from the external environment through ion channels in the plasma membrane [24–26], and second, Ca^2+^ from intracellular Ca^2+^ stores can enter the cytoplasm through the PI-PLC/IP_3_-mediated pathway [27]. In the PI-PLC/IP_3_-mediated pathway, extracellular signals can activate phospholipase C (PLC) and cause an increase in IP_3_, leading to the release of Ca^2+^ from intracellular Ca^2+^ stores [28, 29]. A mean to drastically increase cytosolic Ca^2+^ concentrations in *T. reesei*, however, remains obscure.

Cell permeability plays an important role in substance exchange between the cell and its surrounding medium [30]. Several methods have been developed to artificially overcome the bottleneck presented by cell permeability, either partially or completely, to release massive amounts of target products into the medium, such as permeabilization by organic solvents [30, 31]. Zhong et al. [31] demonstrated that dimethyl sulfoxide (DMSO) treatment increases the permeability of *Panax notoginseng* cells. Takeshige et al. [32] illustrated the use of toluene to permeabilize *Saccharomyces cerevisiae* cells. Permeabilization increases extracellular protein secretion [33, 34]. Bao et al. [33] revealed higher extracellular production of protein with the use of Triton X-100 in culture medium, possibly because of the influence of Triton X-100 on cell permeability or integrity of the cell wall. Stranks et al. [35] also suggested that membrane permeability may be involved in cellulase synthesis in *Myrothecium verrucaria*. It remains unknown, however, if permeabilization can improve cellulase production in *T. reesei*.

*N*,*N*-dimethylformamide (DMF) is a common organic solvent, as it is used extensively as a versatile solvent in large industrial quantities and especially in biological experiments as a solvent for a variety of water-insoluble substrates [36]. In assays using 1,2-bis(*o*-aminophenoxy) ethane-*N*,*N*,*N*′,*N*′-tetraacetic acid (BAPTA), a putative intracellular Ca^2+^ chelator, to investigate the possibility that calcium signaling participates in regulation of cellulase production in *T. reesei* [18], we used DMF as a BAPTA solvent. We found that addition of DMF alone to the medium remarkably induced cellulase production in *T. reesei* Rut-C30. This induction, however, no longer occurred when the versatile solvent DMSO was used instead of DMF.

In the present study, the wild-type strain QM6a was used to study the ability of DMF to induce cellulase production by *T. reesei* and to determine the underlying mechanism. According to the results of a transcriptome analysis, the role of cytosolic Ca^2+^ and the calcium signal transduction pathway in response to DMF in *T. reesei* was deduced. LaCl_3_ and EGTA were used to demonstrate DMF-induced cellulase overexpression through Ca^2+^ uptake and Ca^2+^ signaling in *T. reesei*. Use of the *crz1* deletion strain confirmed this pathway. Additionally, we examined the effects of DMF on *T. reesei* cell permeability and activation of cellulase production after combined DMF and Mn^2+^ treatment. These results are essential for understanding the mechanism of calcium signal transduction in the production of enzymes and provide new insights into a novel inducer of cellulase.

## Results

### Chance discovery of an increase in cellulase activity with the addition of DMF

DMF has been widely used in large-quantity industries as a versatile solvent to produce solutions of water-insoluble substrates. BAPTA, a putative intracellular Ca^2+^ chelator, is only slightly soluble in water. Previously, we used DMF as a solvent to produce a 100×-concentrated stock solution of BAPTA to investigate Mn^2+^ induction of cellulase production [18]. The stock was added to a *T. reesei* culture at a final concentration of 1% (v/v). There was also 1% v/v DMF present in the culture. Serendipitously, we discovered that the independent addition of 1% v/v DMF to the culture medium remarkably increased cellulase production in a cellulase hyper-producing strain of *T. reesei* (Rut-C30) when assessing its effect as a negative control for BAPTA.

To further examine these effects of DMF, the same weight of pre-cultured Rut-C30 mycelia was transferred to fresh liquid minimal medium (MM) containing 1% Avicel as the sole carbon source supplemented with 0 or 1% DMF. CMCase or *p*NPCase activity was directly measured as an indicator of cellulase activity. The results showed that the presence of 1% DMF drastically increased cellulase production: CMCase and *p*NPCase activities were 4.58 ± 0.19 and 0.32 ± 0.02 IU/mL, respectively, which were approximately 2.2 times as high as those of the control (2.12 ± 0.19 and 0.14 ± 0.01 IU/mL, respectively). The mechanism by which DMF increased cellulase activity to this extent, however, was puzzling. To test if other functionally organic compounds identical to DMF also increased cellulase production, we used another versatile solvent, DMSO, instead of DMF to make a stock solution. DMSO alone, however, did not increase cellulase activity to the degree that DMF did (see Additional file 1: Figure S1).

We first found that 1% DMF remarkedly increased cellulase production by *T. reesei* Rut-C30. DMF appeared to be an excellent inducer of double cellulase production in Rut-C30. This induction effect and the mechanism by which DMF regulates cellular metabolism had not been explored. Therefore, we performed additional experiments to explore these important areas.

### DMF was confirmed to increase cellulase production in *T. reesei*

**Fig. 1:**
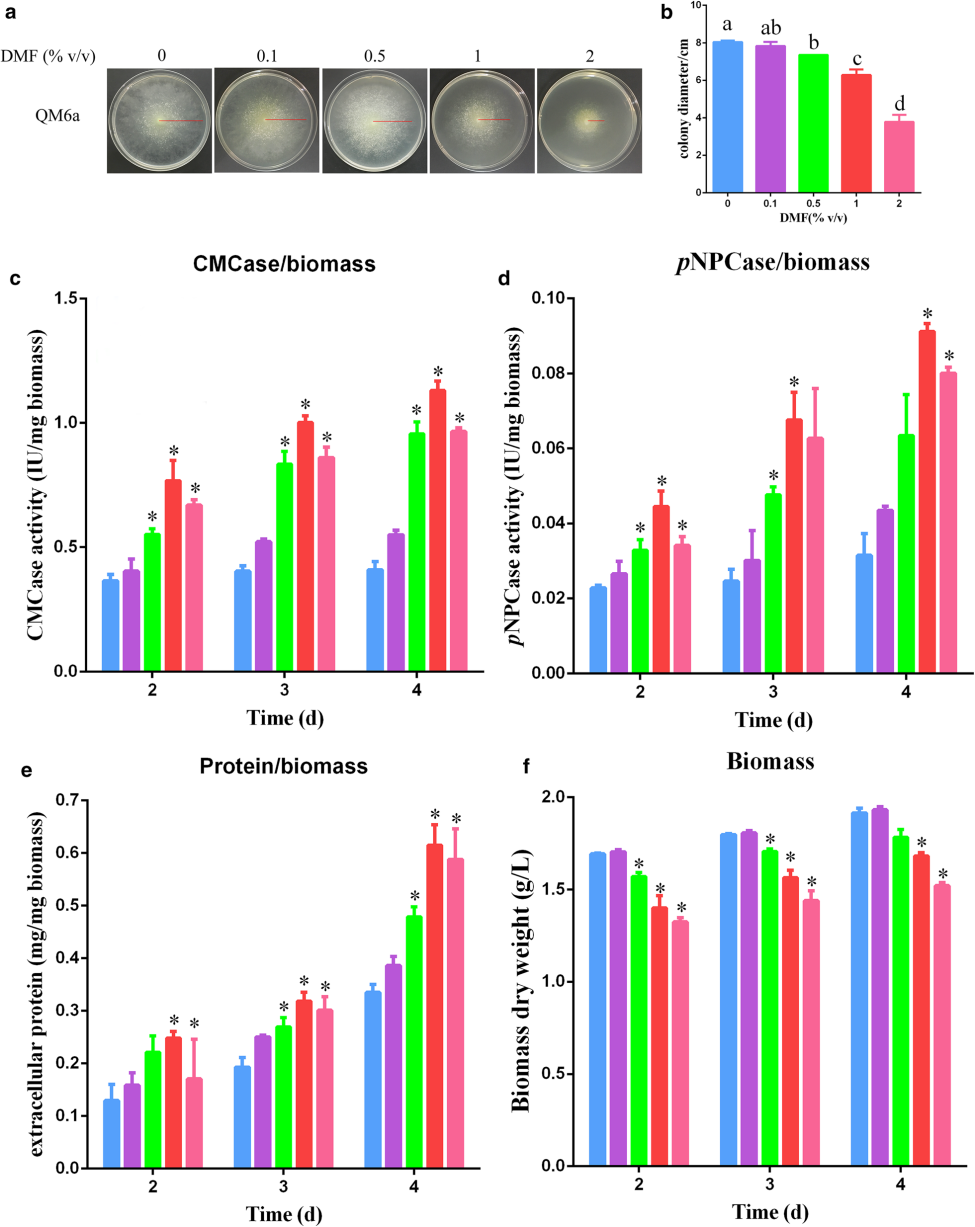
Effects of DMF on hyphal growth and protein production in *T. reesei* QM6a. **a** Hyphae growth of *T. reesei* QM6a on plates. DMF was added at final concentrations of 0, 0.1, 0.5, 1, and 2% (v/v). **b** Colony diameters. **c**–**f** The effects of different concentrations of DMF (final concentration 0, 0.1, 0.5, 1, and 2%) on CMCase/biomass activity (**c**), *p*NPCase/biomass activity (**d**), total protein concentrations (**e**), and biomass (**f**) of *T. reesei* QM6a. Different letters indicate significant differences between the columns (*p* < 0.05, Duncan’s multiple-range test). Blue bar, adding 0% (v/v) DMF; purple bar, adding 0.1% (v/v) DMF; green bar, adding 0.5% (v/v) DMF; red bar, adding 1% (v/v) DMF; pink bar, adding 2% (v/v) DMF. Values are the mean ± SD of results from three independent experiments. Asterisks indicate a significant difference compared to the untreated strain (*p* < 0.05, Student’s *t* test)

To systematically investigate the induction of cellulase production in *T. reesei* by DMF, the wild-type strain QM6a was used. *T. reesei* QM6a was cultured on MM plates supplemented with DMF at concentrations of 0, 0.1, 0.5, 1, and 2% (v/v), with 2% (w/v) glucose provided as the sole carbon source. The mycelium morphology of *T. reesei* after the addition DMF is shown in Fig. 1a and in Additional file 2: Figure S2. The red line represents mycelium morphologies throughout the 4 days of culture. There was no significant difference in hyphal growth between the cultures with 0 and 0.1% DMF; however, at concentrations of 0.5, 1, and 2%, DMF significantly inhibited hyphal growth (Fig. 1b). This was in agreement with the results of biomass production in MM liquid culture (Fig. 1f), which demonstrated significant inhibition of growth upon increasing DMF concentrations, especially in *T. reesei* QM6a treated with 2% DMF.

The same weights of pre-cultured *T. reesei* QM6a mycelia were transferred to fresh liquid MM containing 1% (w/v) Avicel as the sole carbon source supplemented with different concentrations of DMF (0, 0.1, 0.5, 1, and 2%). The *p*NPCase and CMCase activities and total extracellular protein concentrations of the strains are summarized in Fig. 1c–e. There were no significant differences in cellulase activities of the strains treated with 0.1% DMF and those that were untreated; however, the cellulase activities of strains supplemented with 0.5–2% DMF were significantly increased compared to those of the control without DMF treatment. In particular, 1% DMF led to a drastic increase in cellulase production and protein secretion, which was approximately 1.7–2.8 times that of the control.

The above results indicated that 0.5–2% DMF could increase cellulase production in *T. reesei*. This implied that DMF may serve as an inducer of cellulase production in *T. reesei* wild-type QM6a and hyper-cellulolytic mutant Rut-C30. We considered 1% DMF the optimal concentration to enhance cellulase production and total protein secretion while not severely hindering growth, and we used this concentration for further studies.

### DMF increases permeability of *T. reesei* mycelia

**Fig. 2:**
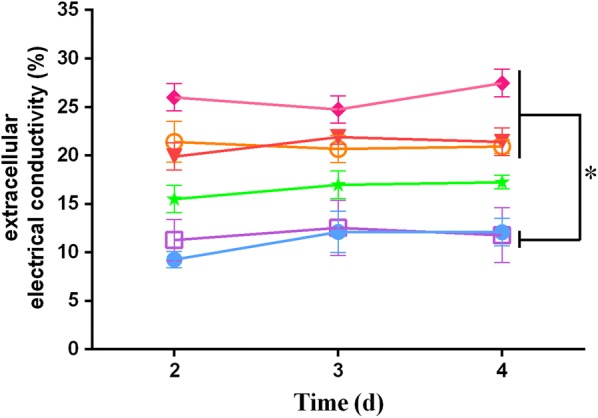
Effects of DMF on cell permeability of *T. reesei* mycelia. The extracellular electrical conductivity of mycelia was measured using an electrical conductivity meter after mycelia were treated with different concentrations of DMF (final concentrations of 0, 0.1, 0.5, 1, and 2%) and 1% (v/v) DMSO. Values are the mean ± SD of the results from three independent experiments. Filled round, adding 0% (v/v) DMF; blank square, adding 0.1% (v/v) DMF; five-pointed star, adding 0.5% (v/v) DMF; triangle, adding 1% (v/v) DMF; diamond, adding 2% (v/v) DMF; blank round, adding 1% (v/v) DMSO. Asterisks indicate a significant difference compared to the untreated strain at all time points examined (*p* < 0.05, Student’s *t* test)

Some investigators [30–32] have shown that organic stimulators affect cell permeability. An increase in cell permeability enhances extracellular protein secretion [33–35]. To investigate the effects of various concentrations of DMF on cell permeability in *T. reesei* mycelia, the extracellular electrical conductivity of mycelia was measured using an electrical conductivity meter according to the methods described in Yan et al. [37]. As is shown in Fig. 2, when 0.5–2% DMF was added, the extracellular electrical conductivity of mycelia increased markedly over time compared to that of the control (0% DMF) and mycelia exposed to 0.1% DMF, with a constant increase in hyphal cell permeabilization and subsequent leakage of inclusion with 0.5–2% DMF treatment. This increase in mycelium permeabilization agrees with the significant enhancement of cellulase production observed after 0.5–2% DMF treatment. This remarkable increase in cellulase induction by DMF did not, however, occur with DMSO treatment, even though DMSO also increases mycelium permeabilization. DMSO improved cellulase activity by only 20–30% compared to that observed in the absence of DMSO (see Additional file 1: Figure S1). Therefore, underlying mechanisms other than increased permeabilization must participate in DMF-induced cellulase production.

### DMF induces cellulase gene overexpression in *T. reesei*

To determine if the effects of DMF on cellulase production are due to enhanced protein secretion or also the induction of cellulase gene transcription, the expression levels of four main cellulase genes (*egl1* encoding endoglucanase I, *egl2* encoding endoglucanase II, *cbh1* encoding cellobiohydrolase I, and *cbh2* encoding cellobiohydrolase II) and the key transcriptional activator of cellulases, *xyr1*, after 0 and 1% DMF supplementation were compared by reverse transcription quantitative PCR (RT-qPCR). After 24 to 72 h incubation with 1% DMF, the transcriptional levels of the four main cellulase genes increased significantly to levels approximately 1.5- to 3.5-fold those of the 0% DMF control (see Additional file 3: Figure S3). These results verified that the upregulation of cellulase activity (Fig. 1c–e) is mainly due to the induction of cellulase gene transcription. The expression of *xyr1*, however, was not significantly stimulated by DMF exposure for 24 to 72 h (data not shown), suggesting that DMF-induced cellulase gene overexpression occurs through other unknown mechanisms rather than through the activation of the key factor *xyr1*. The expression levels of *cbh1* and *egl1* were used to represent cellulase gene expression in further studies.

### Whole transcriptome shotgun sequencing (RNA-seq) provides new insights into cellulase overexpression induced by DMF

To gain insight into the mechanism by which DMF induces cellulase gene overexpression, we compared the two transcriptomes of *T. reesei* QM6a cultured with 0 and 1% DMF (liquid MM containing 1% Avicel as the sole carbon source at 28 °C and 200 rpm). Two biological replicates of each condition were submitted for RNA sequencing using an Illumina HiSeq X Ten. Processing of individual samples was successful (46,006,536–57,345,532 reads, without a significant difference between the replicates) (see Additional file 4: Table S1). Following sequence quality control, the reads were mapped to a *T. reesei* genome (http://genome.jgi.doe.gov/Trire2/Trire2.home.html) with 93.17–94.95% coverage (see Additional file 4: Table S1). There was a high correlation (Pearson correlation, *r*^2^ ≥ 0.78) between the two biological replicates of each condition used in the transcriptional analysis (Additional file 5: Figure S4).

Next, we searched for those transcripts that differed by an adjusted fold change of ≥ 2 or unadjusted fold change ≤ 1/2 and false discovery rate (adjusted *p* value) < 0.01 with 0 and 1% DMF supplementation. This resulted in the retrieval of 102 genes that were differentially expressed following 1% DMF treatment compared to control (no DMF addition). Of these, 81 were upregulated and 21 were down-regulated (see Additional file 6: Figure S5a; Additional file 7: Table S2). Then, functional categorization of the 102 up- and down-regulated genes was performed using Gene Ontology (GO) terms (see Additional file 6: Figure S5b). The enrichment analysis showed that in the presence of DMF, most of the differentially expressed genes were related to carbohydrate metabolism, extracellular region, cellulose binding, and hydrolase activity.

In total, 13 cellulose degradation-related genes were significantly upregulated in the presence of 1% DMF (Table 1). Two main cellobiohydrolase-encoding genes (*cbh1* and *cbh2*; IDs: 123989 and 72567), four endoglucanase-encoding genes (IDs: 122081, 120312, 49976, and 54242), including two main endoglucanase genes (*egl1* and *egl2*), one beta-glucosidase-encoding gene (*cel3b*; ID: 121735), and a xylanase-encoding gene (*xyn3*; ID: 120229) were upregulated in response to 1% DMF supplementation. Additionally, transcription of accessory protein-encoding genes was noticeably elevated after 1% DMF treatment, including that of the swollenin gene *swo1* (ID: 123992) and *cip2* (ID: 123940) [5]. The transcriptome data agreed with the cellulase activity and qPCR results (Fig. 1c, d and Additional file 3: Figure S3). These results revealed that 1% DMF treatment significantly upregulated transcription of a set of cellulose degradation-related genes.

**Table 1:** Log_2_-fold changes in major cellulose degradation-related gene transcripts with the addition of DMF

Gene ID	Annotation	Log_2_ fold change (DMF^a^/WT^b^)	Regulate
Trire2_123989	Cellobiohydrolase CBH1/Cel7a	3.16	Up
Trire2_72567	Cellobiohydrolase CBH2/Cel6a	3.55	Up
Trire2_122081	Endoglucanase EGL1/Cel7b	3.82	Up
Trire2_120312	Endoglucanase EGL2/Cel5a	4.05	Up
Trire2_49976	Endoglucanase EGL5/Cel45a	3.78	Up
Trire2_54242	Endoglucanase Cel55a	2.36	Up
Trire2_121735	Beta-glucosidase Cel3b	2.73	Up
Trire2_120229	Endo-1,4-beta-xylanase XYNIII	3.98	Up
Trire2_123992	Swollenin	3.35	Up
Trire2_69276	Cellulosome enzyme	3.84	Up
Trire2_76210	Glycoside hydrolase	3.16	Up
Trire2_123940	Cellulose-binding protein CIP2	3.72	Up
Trire2_71554	Cellulase	4.92	Up

^a^DMF, gene expression level in parental strain QM6a with 1% DMF supplementation

^b^WT, gene expression level in parental strain QM6a with 0% DMF supplementation

Additionally, based on transcription profiling, we speculated on the mechanism underlying DMF induction of cellulase gene overexpression. The gene *plc*-*e* (Trire2:21960; GenBank accession number: ABG20593), which encodes a phospholipase C protein that generates inositol-1,4,5-trisphosphate (IP3) to regulate calcium release from intracellular pools [38], was remarkably upregulated (approximately four times that of the control) in DMF-induced strains (see Additional file 8: Table S3). The transcriptional levels of *plc*-*e* were also analyzed by RT-qPCR (see Additional file 8: Table S3). The qPCR results agreed with those of the whole transcriptome shotgun sequencing analysis. Further, deletion of *plc*-*e* effectively weakened the induction effect of DMF on cellulase production (see Additional file 9). These results suggested that DMF may affect cytosolic Ca^2+^ levels and that Ca^2+^ signaling may contribute to the induction of cellulase genes by DMF.

### DMF induces cytosolic Ca^2+^ accumulation and calcium signal transduction in *T. reesei*

**Fig. 3:**
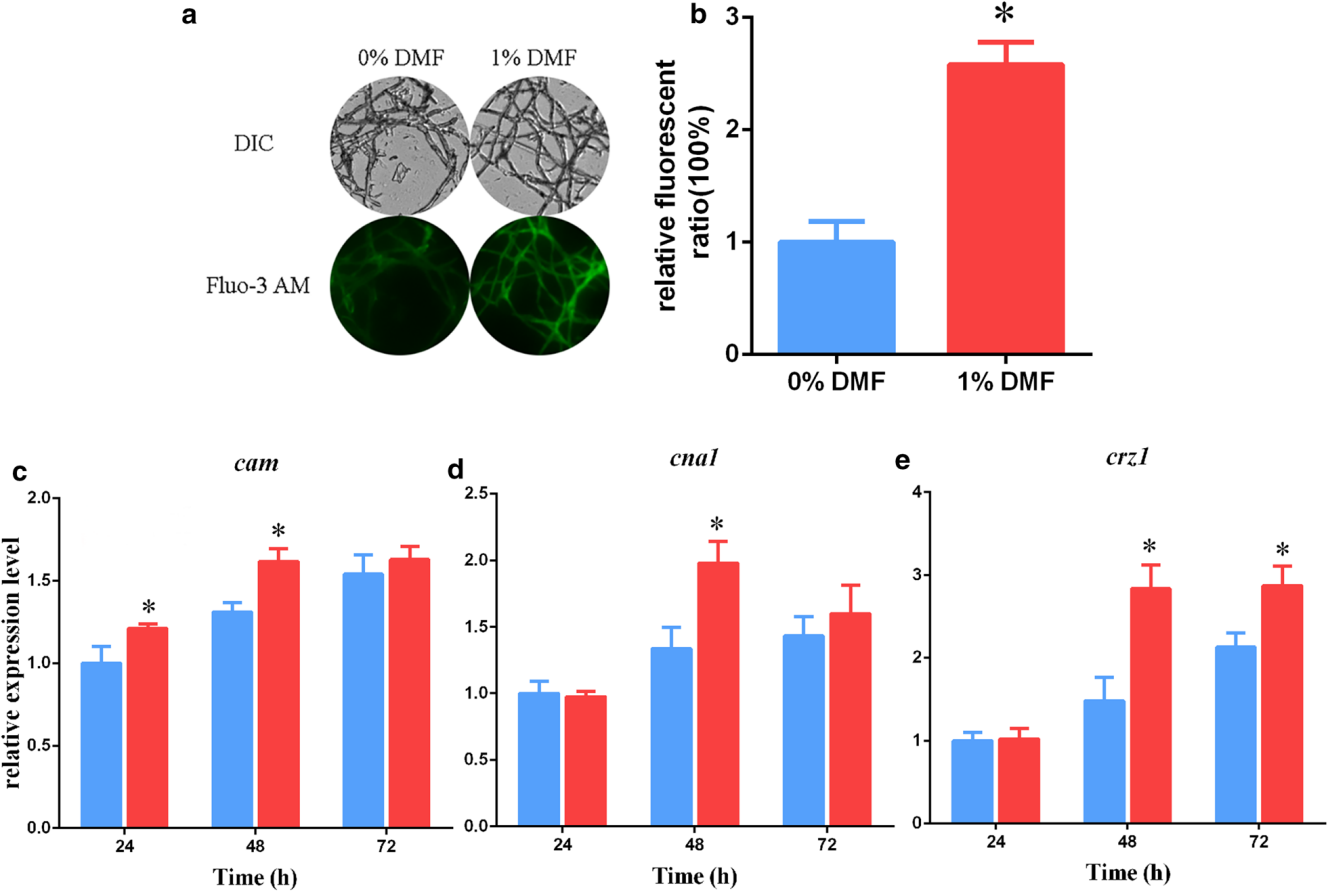
Cytosolic Ca^2+^ level and calcium signaling increases after DMF addition. **a** Cytosolic Ca^2+^ levels determined by Ca^2+^ fluorescent probe Fluo-3 AM. *T. reesei* QM6a was cultured in liquid Mandels’ medium for ~ 32 h, transformed to fresh MM with 0% or 1% DMF, and then cultivated for 48 to 60 h. For detection, 50 μM Fluo-3 AM was used, with the intensity of fluorescence monitored using automatic inverted fluorescence microscopy. Green fluorescence represents free cytosolic Ca^2+^. DIC, differential interference contrast. **b** Comparative fluorescence ratio of cytosolic Ca^2+^ levels. The *x*-axis represents the different DMF final concentrations, and the *y-*axis represents the Ca^2+^ fluorescence ratios measured by CLSM. **c**–**e** The effects of DMF on the transcriptional levels of calcium signaling-related genes *cam* (**c**), *cna1* (**d**), and *crz1* (**e**) in *T. reesei* QM6a. Blue bar, no DMF was added to the medium; red bar, 1% (v/v) DMF was added to the medium. Values are the mean ± SD of the results from three independent experiments. Asterisks indicate significant differences from untreated strains (*p* < 0.05, Student’s *t* test)

Based on the results of transcriptional profiling, we hypothesized that DMF affects cytosolic Ca^2+^ levels. To investigate this hypothesis, Fluo-3 AM fluorescent dye, which emits green fluorescence after crossing the cell membrane and binding to cytosolic Ca^2+^, was used to determine intracellular Ca^2+^ concentrations [18, 39]. As shown in Fig. 3a, with 1% DMF treatment, green fluorescence intensity was stronger in the QM6a cells on the second day than that observed in the control (no DMF addition), confirming a intracellular Ca^2+^ concentration increase after 1% DMF treatment. Fluorescence analysis revealed that after 1% DMF supplementation, the Ca^2+^-activated fluorochrome level was 2.5-fold that of the control (Fig. 3b). To more thoroughly investigate the changes in intracellular Ca^2+^, ICP-MS was used as previously described [18]. The intracellular Ca^2+^ value was 108.74 ± 5.44 μmol/g biomass after 1% DMF addition, and this was approximately 1.70-fold that observed in controls (64.12 ± 4.19 μmol/g biomass). These results indicate that DMF induced an increase in the level of cytosolic Ca^2+^. Additionally, DMSO could not induce cytosolic Ca^2+^ accumulation, as the intracellular Ca^2+^ value was 68.31 ± 4.46 μmol/g biomass after 1% DMSO addition, which was not significantly different from the control value of 64.12 ± 4.19 μmol/g biomass. This may be the major reason for the observed difference in cellulase production induced by DMSO and DMF.

Cytosolic Ca^2+^ is one of the foremost second messengers that induce calcium signaling to trigger downstream events [40, 41]. Our previous work involving Mn^2+^ elicitation showed that increased levels of cytosolic Ca^2+^ can trigger calcium signal transduction pathways and induce cellulase expression in *T. reesei* [18]. The mechanism of DMF induction of cellulase production may be similar to that of Mn^2+^ stimulation [18]. To test our hypothesis, the expression levels of three calcium signaling genes (*cam*, *cna*, and *crz1*) were quantitatively determined by RT-qPCR after treatment with 1% DMF. As illustrated in Fig. 3c–e, the transcription of these genes was upregulated after the addition of DMF, which is consistent with the observed increase in cytosolic Ca^2+^ concentrations.

These results indicated that DMF can induce a cytosolic Ca^2+^ burst that activates the calcium signal transduction pathway to regulate cellulase production in *T. reesei*.

### The cytosolic Ca^2+^ burst induced by DMF mediates overexpression of cellulase

Our previous study revealed the effects of Mn^2+^ induction of cytosolic Ca^2+^ on cellulase production in *T. reesei* [18]. To address if the cytosolic Ca^2+^ burst induced by DMF mediates enhanced cellulase production, we used EGTA (a putative extracellular Ca^2+^ chelator) to chelate extracellular Ca^2+^ and LaCl_3_ (a plasma membrane Ca^2+^ channel blocker) to prevent the influx of external Ca^2+^ [42].

**Fig. 4:**
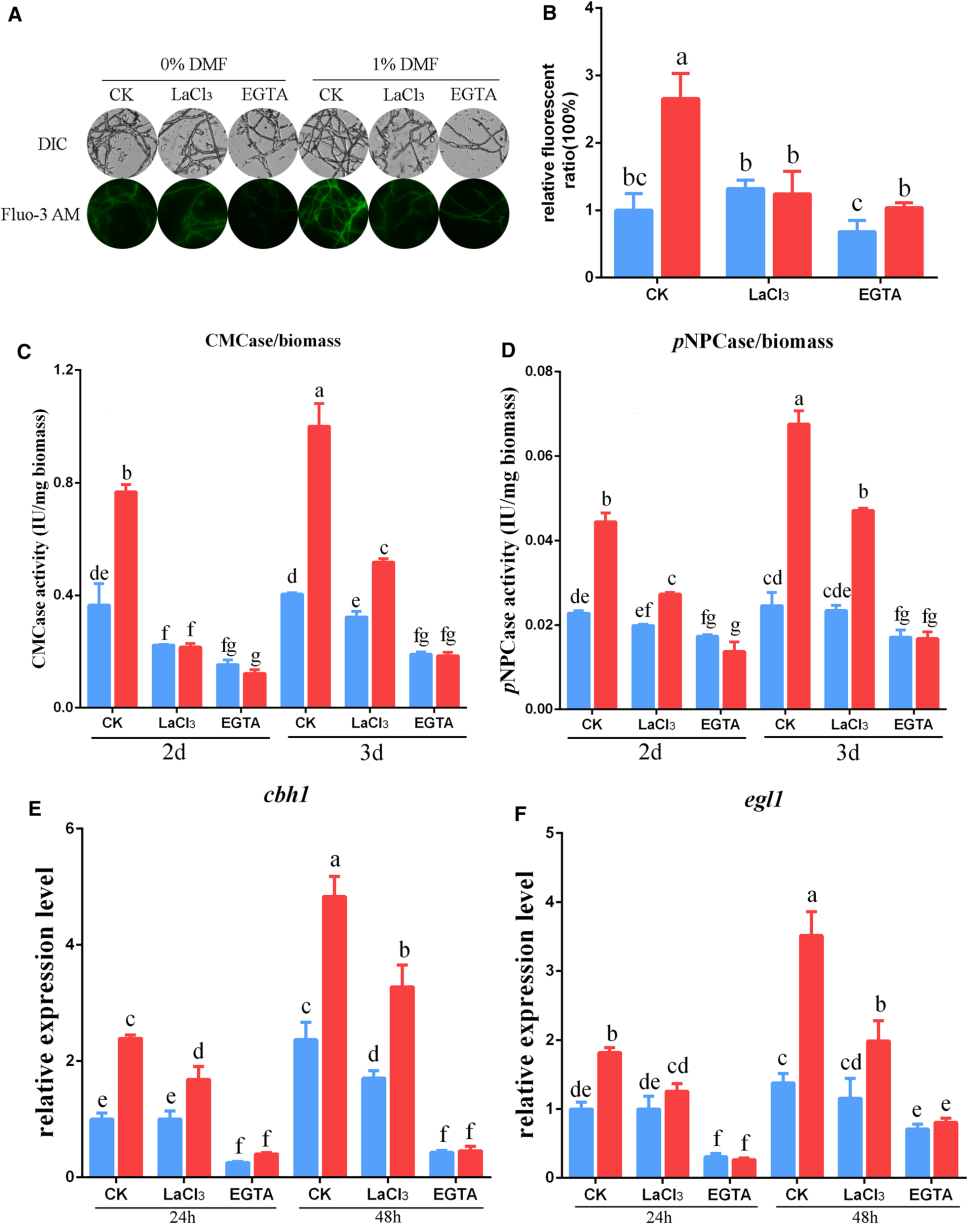
Effect of Ca^2+^ inhibitors on cytosolic Ca^2+^ concentrations and cellulase production after DMF addition. **A** Fluorescence indicating LaCl_3_ and EGTA effects on the cytosolic Ca^2+^ burst induced by DMF. *T. reesei* QM6a was cultured in liquid Mandels’ medium for ~ 32 h, transformed to fresh MM with 0% or 1% DMF and 0 or 5 mM LaCl_3_/EGTA, and cultivated for 48 to 60 h. For detection, 50 μM Fluo-3 AM was used, and fluorescence intensity was monitored using automatic inverted fluorescence microscopy. Green fluorescence represents free cytosolic Ca^2+^. DIC, differential interference contrast. CK, not treated with inhibitors LaCl_3_/EGTA. **B** Comparative fluorescence ratios indicating LaCl_3_ and EGTA effects on the cytosolic Ca^2+^ burst induced by DMF. The *x-*axis represents different treatments with DMF and inhibitors, and the *y*-axis represents Ca^2+^ fluorescence ratios measured by CLSM. **C**, **D** CMCase/biomass activity (**C**) and *p*NPCase/biomass activity (**D**) were examined after *T. reesei* QM6a was cultured in medium with different DMF and inhibitor treatments. **E**, **F** The expression levels of *cbh1* (**E**) and *egl1* (**F**) in *T. reesei* QM6a were analyzed after culture in medium with various DMF and inhibitor treatments. Blue bar, no DMF was added to the medium; red bar, 1% (v/v) DMF was added to the medium. Values are the mean ± SD of the results from three independent experiments. Different letters indicate significant differences between the columns (*p* < 0.05, Duncan’s multiple-range test)

When the *T. reesei* strains were treated with LaCl_3_ or EGTA, the fluorescence intensity of mycelia markedly decreased compared with that of mycelia exposed to 1% DMF in the absence of Ca^2+^ inhibitors (Fig. 4A). The quantitative results indicated that the addition of LaCl_3_ or EGTA led to a greater loss of cytosolic Ca^2+^ (53% and 61% reductions, respectively) than observed in the no inhibitor control after exposure to 1% DMF (Fig. 4B). These results indicated that the cytosolic Ca^2+^ burst induced by 1% DMF was effectively blocked by adding LaCl_3_ or EGTA.

We also found that with the addition of 0% DMF, LaCl_3_ or EGTA did not noticeably decrease cytosolic Ca^2+^ in comparison with observations of the no inhibitor control (Fig. 4B). This may have contributed to relatively low levels of intracellular Ca^2+^ in *T. reesei* QM6a when stimulants such as DMF and Mn^2+^ were not added. Fluo-3 AM fluorescent dye did not allow us to accurately distinguish between these low baseline intracellular Ca^2+^ concentration; however, 1% DMF led to a cytosolic Ca^2+^ burst in *T. reesei*. Given this, we observed significant differences in fluorescence intensities between DMF-induced *T. reesei* with and without the addition of Ca^2+^ inhibitors.

To assess if blocking the cytosolic Ca^2+^ burst could hinder the overexpression of cellulase induced by DMF, we analyzed CMCase and *p*NPCase activities and transcriptional levels of the key cellulase genes *cbh1* and *egl1*. As shown in Fig. 4C–F, the addition of EGTA led to significant reductions in CMCase and *p*NPCase activities and transcriptional levels of *cbh1* and *egl1* compared to those in a no EGTA control with 0 or 1% DMF supplementation. Additionally, there was no difference between exposure to 0 and 1% DMF in the context of cellulase gene expression and cellulase activity when EGTA was added (Fig. 4C–F). The increase in cellulase induced by 1% DMF could be totally abolished by adding EGTA. Conversely, LaCl_3_ only caused a slight decrease in cellulase transcription levels and cellulase activity compared to that in the no LaCl_3_ control when no DMF was added (Fig. 4C–F). After 1% DMF supplementation, however, CMCase and *p*NPCase activities and transcriptional levels of the key cellulase genes *cbh1* and *egl1* decreased remarkably when LaCl_3_ was added compared to those observed in the no LaCl_3_ control (Fig. 4C–F). These results indicated that blocking the cytosolic Ca^2+^ burst by LaCl_3_ and EGTA could significantly reduce and even abrogate the effect of DMF induction on cellulase production (Fig. 4).

Overall, these data indicated that DMF induced a cytosolic Ca^2+^ burst by increasing extracellular Ca^2+^ uptake through Ca^2+^ channels, as illustrated by the observation that when extracellular Ca^2+^ was chelated with EGTA or Ca^2+^ channels were blocked by LaCl_3_, the increase in levels of cytosolic Ca^2+^ was attenuated. Overexpression of cellulase, therefore, is mediated by a cytosolic Ca^2+^ burst induced by DMF.

### DMF induces cellulase overexpression via calcium signaling

The calcineurin-responsive zinc finger transcription factor 1, *crz1*, plays an essential role in calcium signal transduction as a terminal protein in the calcium signaling cascade. Earlier studies showed that CRZ1 induced cellulase production at the transcriptional level [13, 18]. To further clarify the role of calcium signaling in DMF induction of cellulase overexpression, we investigated the effects of DMF induction on cellulase production in the *crz1* deletion strain Δ*crz1*.

**Fig. 5:**
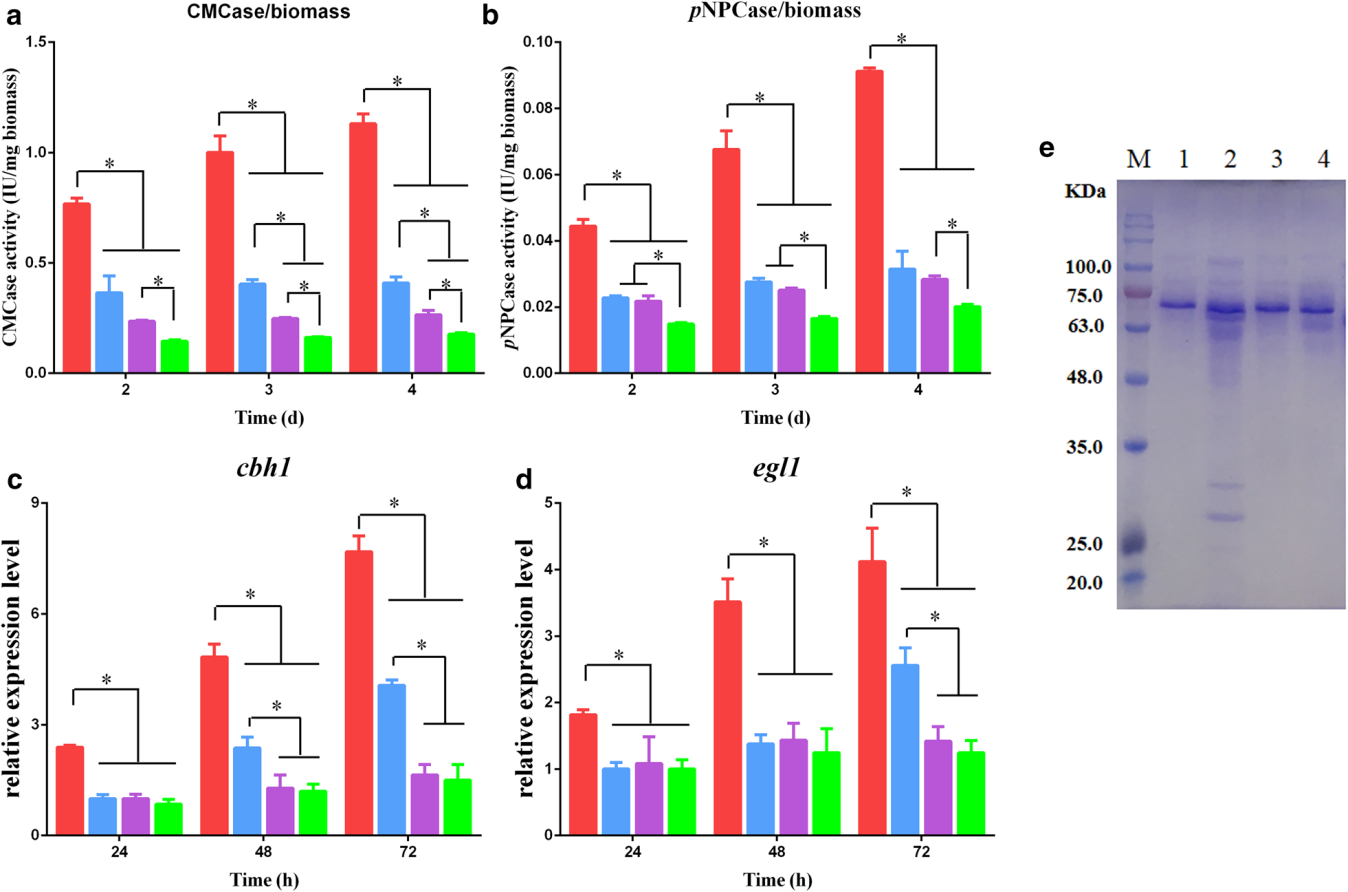
Effect of CRZ1 on DMF-induced cellulase overexpression. **a**, **b** CMCase/biomass activity (**a**) and *p*NPCase/biomass activity (**b**) of *T. reesei* QM6a and Δ*crz1* strains supplemented with 0% or 1% DMF. **c**, **d** The expression levels of *cbh1* (**c**) and *egl1* (**d**) in *T. reesei* QM6a and Δ*crz1* strains cultured in medium supplemented with 0% or 1% DMF. Red bar, adding 1% (v/v) DMF in *T. reesei* QM6a; blue bar, adding 0% (v/v) DMF in *T. reesei* QM6a; purple bar, adding 1% (v/v) DMF in Δ*crz1*; green bar, adding 0% (v/v) DMF in Δ*crz1*. **e** SDS-PAGE of the secretomes in supernatants. *T. reesei* QM6a and Δ*crz1* were cultured in liquid Mandels’ medium for ~ 32 h. Then, mycelia were transferred to fresh Mandels’ medium with 0% or 1% DMF and cultivated for 72 h. Two cultures of each strain with 0% or 1% DMF added were collected by centrifugation and diluted similar to the proper protein concentration for SDS-PAGE. Lines 1 and 2 represent supernatants from the QM6a culture supplemented with 0% or 1% DMF, respectively. Lines 3 and 4 represent supernatants from the Δ*crz1* culture supplemented with 0% or 1% DMF, respectively. Values are the mean ± SD of the results from three independent experiments. Asterisks indicate significant differences (*p < 0.05, Student’s *t* test)

In response to 1% DMF induction, the Δ*crz1* strain showed only an approximately 30% increase in CMCase and *p*NPCase activities compared to those observed after no DMF induction (Fig. 5a, b), and this was considerably less than the 100–200% enhancement observed in the parental strain QM6a after DMF induction. This indicated that DMF induction was remarkably reduced in the Δ*crz1* mutant. Results of an SDS-PAGE analysis (Fig. 5e) of extracellular proteins secreted by QM6a and Δ*crz1* agreed with the cellulase activity data above. Additionally, the marked increase in levels of *cbh1* and *egl1* transcription induced by DMF, observed in the parental strain QM6a, was abolished in the *crz1* deletion strain at all time points examined (Fig. 5c, d). These results indicated that the increase in cellulase production induced by DMF is reduced in the *crz1* deletion strain. These results also indicated that calcium signals participated in and play a dominant role in DMF induction of cellulase overexpression in *T. reesei*.

### Combining DMF and Mn^2+^ can significantly enhance cellulase production in *T. reesei* Rut-C30

**Fig. 6:**
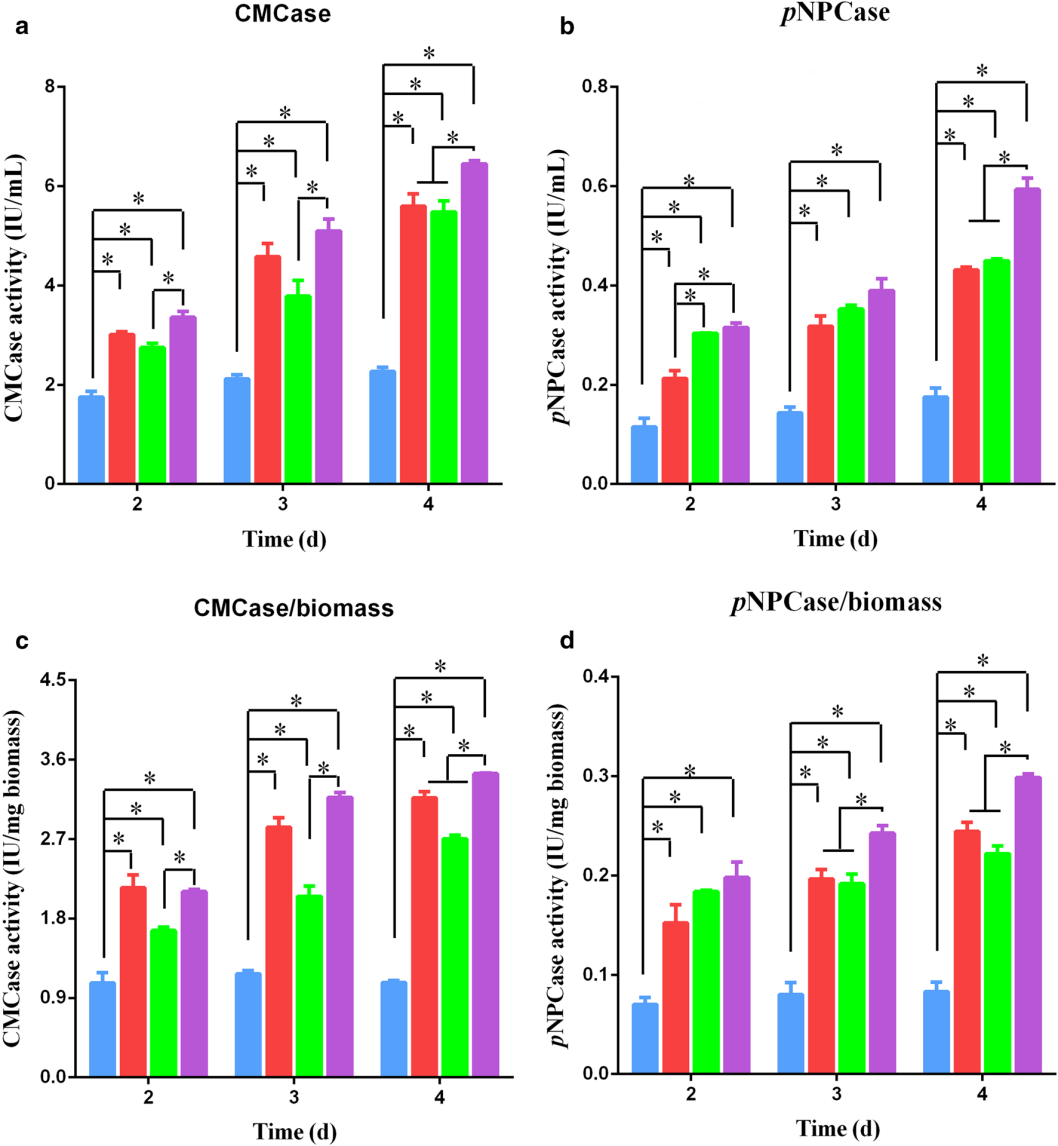
Effects of combined DMF and Mn^2+^ on cellulase production in *T. reesei* Rut-C30. **a**–**d** CMCase activity (**a**), *p*NPCase activity (**b**), CMCase/biomass activity (**c**) and *p*NPCase/biomass activity (**d**) of *T. reesei* Rut-C30 supplemented with 1% DMF or (and) 10 mM MnCl_2_. Blue bar, no addition; red bar, 1% (v/v) DMF was added to the medium; green bar, 10 mM MnCl_2_ was added to the medium; purple bar, 1% (v/v) DMF and 10 mM MnCl_2_ was added to the medium. Values are the mean ± SD of the results from three independent experiments. Asterisks indicate significant differences (*p < 0.05, Student’s *t* test)

In our previous study [18], we found that a biologically relevant extracellular Mn^2+^ concentration markedly stimulated cellulase production in *T. reesei* Rut-C30, a well-known industrial workhorse for cellulase production, via calcium channels and calcium signaling in a manner similar to that induced by DMF. To address if combining DMF and Mn^2+^ could further improve cellulase production by Rut-C30, the effects of DMF and Mn^2+^ cross-talk on cellulase production of *T. reesei* Rut-C30 were investigated. As shown in Fig. 6, with exposure to 1% DMF or 10 mM Mn^2+^, CMCase and *p*NPCase activities were markedly increased in *T. reesei* Rut-C30 compared to those when no treatment was administered. Additionally, with simultaneous 1% DMF and 10 mM Mn^2+^ supplementation, CMCase and *p*NPCase activities were even higher than with individual treatments of either 1% DMF or 10 mM Mn^2+^ after 4 days of cultivation. These results provide insight into the industrial applicability of DMF for cellulase production.

## Discussion

In this study, we serendipitously discovered that treatment with 1% DMF significantly increased cellulase production (approximately two times more) in both *T. reesei* wild-type strain QM6a and the hyper-cellulolytic mutant Rut-C30. When the *T. reesei* strains were cultured in a nutrient-rich fermentation medium (glucose 5 g/L; lactose 37 g/L; corn syrup 27 g/L; (NH_4_)_2_SO_4_ 5 g/L; KH_2_PO_4_ 6 g/L; CaCl_2_ 0.5 g/L; MgSO_4_ 1 g/L; FeSO_4_**·**7H_2_O 5 mg/L; MnSO_4_**·**1H_2_O 1.6 mg/L; ZnSO_4_**·**7H_2_O 1.4 mg/L; CoCl_2_**·**6H_2_O 2 mg/L; pH 5.5), cellulase production after exposure to 1% DMF could be significantly improved to approximately three–five times that of mycelia with no DMF supplementation. These results indicated that biologically relevant extracellular DMF concentrations can stimulate cellulase production in *T. reesei*. To the best of our knowledge, the effect of DMF on cellulase production has not been reported previously. Our research will aid in enhancing the induction efficiency of industrial cellulase production. Known cellulase inducers of *T. reesei*, including cellulose, cellobiose, lactose, and sophorose, can be used as the sole carbon source; however, *T. reesei* strains cannot grow with DMF as the sole carbon source. This is the primary difference between DMF and known cellulase inducers.

As the cell wall and plasma membrane are the initial cellular barriers, permeabilization is important for the secretion of massive amounts of target products. Our research demonstrated that DMF and DMSO can increase the permeability of *T. reesei* mycelia (Fig. 2). The results also indicated that increased permeabilization can improve cellulase production in *T. reesei* and suggest that treatment with organic solvents is an effective strategy for releasing fungal proteins. Despite the fact that 1% DMSO increased the permeability of *T. reesei* mycelia in a manner similar to DMF, however, DMSO cannot increase cellulase activities in a manner similar to DMF. This implies that permeabilization is not the key factor for DMF-induced cellulase production in *T. reesei*.

In our transcriptome analyses, we found that 1% DMF could significantly upregulate expression of several cellulose degradation-related genes, including four main cellulase-encoding genes and the two auxiliary protein-encoding genes *swo1* and *cip2*. Swollenin, which has a cellulose-specific carbohydrate binding module (CBM) linked to an expansin-like domain [5], exhibits hydrolytic activity with features of both endoglucanases and cellobiohydrolases [43]. Cellulose-induced protein 2 (CIP2), acts on ester linkages between hemicellulose and lignin and is important for efficient degradation of lignocellulose [44]. Remarkably, the expression levels of *xyn3*, which is coordinately expressed with cellulase genes [43], were also upregulated in response to 1% DMF supplementation.

Most importantly, we found by whole transcriptome shotgun sequencing that the transcription levels of the phospholipase C-encoding gene *plc*-*e* were clearly upregulated during DMF induction (Additional file 8: Table S3). Phospholipase C participates in the release of calcium to increase cytosolic Ca^2+^ concentrations. The significantly upregulated *plc*-*e* transcription implied that Ca^2+^ signaling pathways may participate in DMF-induced cellulase overexpression. Results of our study also indicated that 1% DMF increased the level of cytosolic Ca^2+^ in *T. reesei* (Fig. 3a, b). In addition, in our experiments, inhibition of cytosolic Ca^2+^ by LaCl_3_ or EGTA was attenuated and even abolished by DMF induction of cellulase (Fig. 4). Additionally, our study demonstrated that Ca^2+^-activated CRZ1 participated in regulating cellulase gene expression during DMF induction (Fig. 5a–d). Together, these results suggest that Ca^2+^ signaling plays a dominant role in response to DMF induction.

It should be mentioned that *T. reesei* has four phospholipase C homologues with characteristics of eukaryotic phospholipase C proteins; however, one (PLC-E) is most similar to prokaryotic phospholipase C proteins [38]. Additionally, signal transduction by PLC-E remains unknown. Further, we found that deletion of *plc*-*e* effectively weakened the induction effect of DMF on cellulase production (see Additional file 9). These results imply that PLC-E is involved in Ca^2+^ signal transduction induced by DMF in *T. reesei*.

**Fig. 7:**
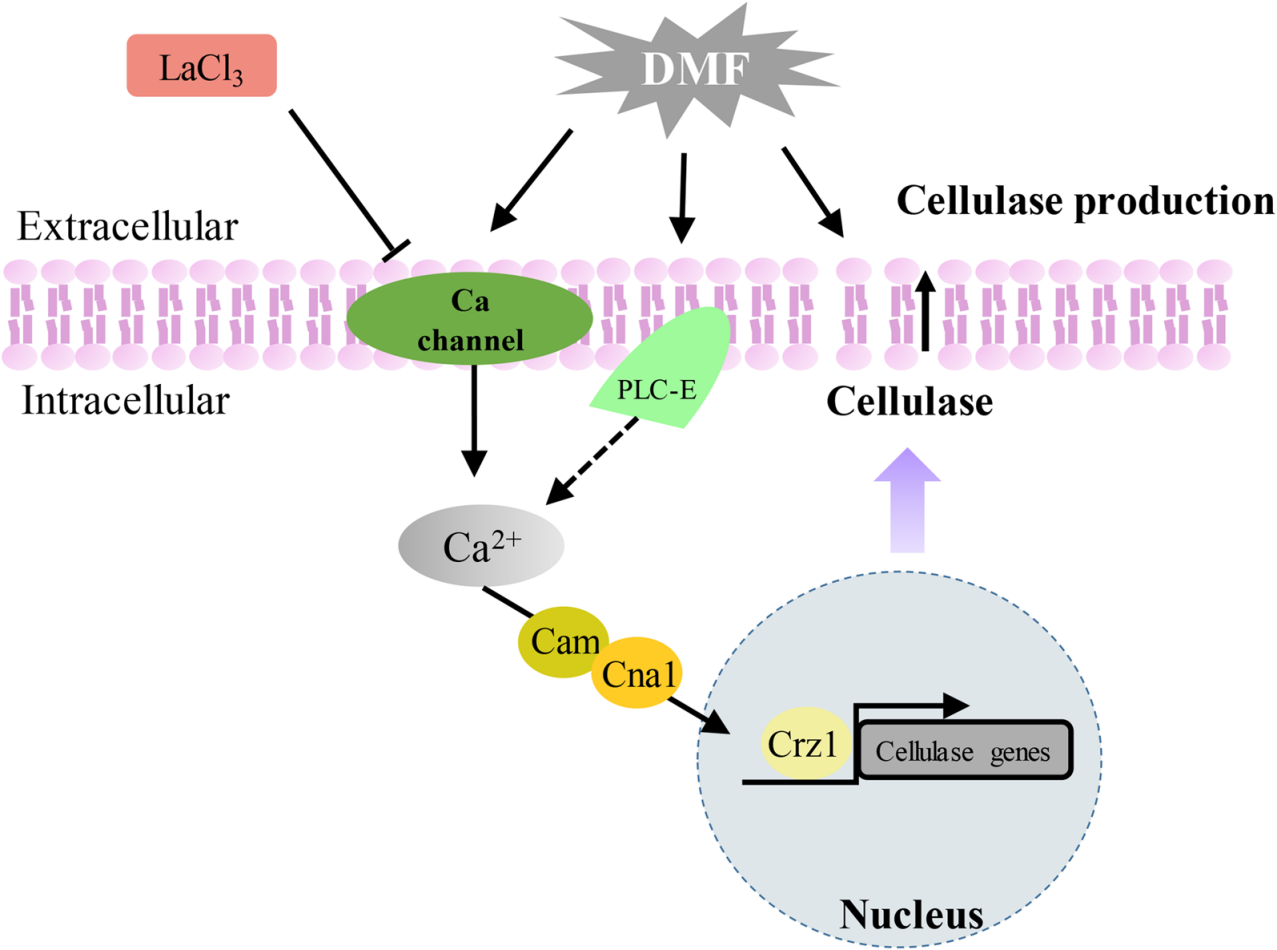
Mechanistic model of DMF induction of cellulase overexpression in *T. reesei*. DMF promotes a cytosolic Ca^2+^ burst that activates calcium signaling, which in turn is required for cellulase overexpression. The effects of adding EGTA and LaCl_3_ suggest that extracellular Ca^2+^ and Ca^2+^ channels are responsible for the cytosolic Ca^2+^ burst and cellulase production induced by DMF. Deletion of *crz1* confirmed that calcium signaling plays a dominant role in DMF-induced cellulase production. Deletion of *plc*-*e* implied that PLC-E is involved in Ca^2+^ signal transduction induced by DMF. Additionally, the cell permeability of *T. reesei* mycelia increased after treatment with DMF, which is beneficial for cellulase secretion. Solid arrows indicate data supported by our own experiments

Additionally, we found that the concentrations of extracellular DMF were almost constant in the tested strains. This suggested that DMF-induced cellulase overexpression by stimulating calcium signaling. Deletion of *crz1* interruption of calcium signaling partially blocked DMF-induced cellulase overexpression but did not abolish it (Fig. 5a, b). CMCase and *p*NPCase activities were, however, still slightly increased by approximately 30% in a Δ*crz1* strain treated with DMF (Fig. 5a, b) in a manner similar to DMSO (Additional file 1: Figure S1). In addition, SDS-PAGE analysis (Fig. 5e) of extracellular proteins secreted by QM6a and Δ*crz1* indicated that DMF can still slightly enhance extracellular cellulase production in Δ*crz1*, but this enhancement is attenuated compared to that in QM6a. These results imply that increased permeability of *T. reesei* cells by DMF is responsible for the slight enhancement of cellulase in Δ*crz1*, where DMF-induced cellulase overexpression via calcium signaling was blocked (Fig. 7). This result suggests that because DMSO cannot affect cytosolic Ca^2+^ levels, Ca^2+^ signaling, rather than cell permeabilization, is the dominant mechanism for DMF induction of cellulase. DMF and Mn^2+^ in combination resulted in greater production of cellulase, indicating that DMF induces cellulase through a mechanism other than the Ca^2+^ signaling of Mn^2+^, such as permeabilization, providing insight into the industrial applicability of DMF for cellulase production.

Lactose, which accumulates as a byproduct of cheese manufacturing or whey processing, is an inexpensive soluble inducer of cellulase production in *T. reesei*, as discovered recently [45–47]. As lactose occurs in nature only in the milk of mammals and is not present in the natural habitat of *T. reesei*, this induction effect is surprising [48]. Similarly, DMF, synthesized for the first time in 1893, is an anthropogenic compound that does not occur in nature and therefore does not exist in the natural habitat of *T. reesei* [49, 50]. In this study, we found that DMF-stimulated cellulase production in *T. reesei*. We hypothesize that the structure of DMF may be similar to unknown natural inducers of cellulase such as lactose, which acts as an inducer due to structural similarity to substances present in the natural habitat of *T. reesei* [48]. In our study, we also treated *T. reesei* with DMSO, another versatile solvent similar to DMF; however, DMSO did not exert a significant effect on cellulase production in *T. reesei*. Therefore, not all versatile solvents can induce cellulase production, and DMF offers a particular advantage in regard to this phenomenon. Further, neither the DMF analog *N*,*N*-dimethylacetamide nor the DMF hydrolysate *N*,*N*-dimethylamine has any significant effect on cellulase overexpression in *T. reesei* (see Additional file 10: Figure S6), while another DMF analog, *N*-methylformamide, which has a similar effect on cellulase production in *T. reesei*, can double cellulase production at a concentration of 1% (see Additional file 10: Figure S6). We speculate that the formyl group of DMF and *N*-methylformamide is an important structure for cellulase induction. In future studies, analogs can be screened as suitable inducer candidates by assaying their effect on cellulase production, similar to the way that isopropyl β-d-1-thiogalactopyranoside (IPTG) came to replace lactose for use in high-throughput protein expression in *Escherichia coli* [51]. Our research provides new insights into the mechanism underlying cellulase induction and sheds light on potential novel inducers of cellulase production.

Additionally, it should be noted that DMF, because it is miscible with water and various organic solvents, is extensively used in many chemical industries [52]. Consequently, DMF is commonly discharged in high concentrations along with industrial effluents [53]. In our study, we found that the addition of DMF effluents could also induce cellulase overexpression in *T. reesei* (unpublished data). These characteristics make DMF wastewater an ideal additive for industrial applications that will facilitate both cellulase overexpression and wastewater reuse.

## Conclusions

In summary, this study provides a putative mechanism for DMF-induced cellulase production in *T. reesei* (Fig. 7). Our results indicate that under this DMF-mediated induction, Ca^2+^ signaling, phospholipase C (PLC-E), and cellular permeability participate in cellulase over-production. First, DMF stimulates a significant increase in levels of cytosolic Ca^2+^ and triggers Ca^2+^-CRZ1 signaling to induce transcription of cellulase genes. Second, DMF enhances cellulase production by increasing the cellular permeability of *T. reesei* mycelia. This study may provide new insights into the mechanism underlying cellulase induction and lead to the discovery of novel inducers for cellulase production. Our research suggests DMF wastewater as an ideal additive in industrial applications, as this will facilitate both cellulase overexpression and wastewater reuse.

## Methods

### Strains and growth conditions

*Escherichia coli* DH5α was used for plasmid amplification. *T. reesei* QM6a (ATCC 13631) and Rut-C30 (ATCC 56765) were used for the studies. *E. coli* was cultured in Luria broth (LB) medium, and Mandels’ medium [54] was used for general fungal culture. All strains of *T. reesei* were maintained on potato dextrose agar (PDA) plates at 28 °C. All strains were cultured in the dark.

Minimal medium (MM) (Urea 0.3 g/L; (NH_4_)_2_SO_4_ 5 g/L; KH_2_PO_4_ 15 g/L; MgSO_4_ 0.6 g/L; CaCl_2_ 0.6 g/l; FeSO_4_**·**7H_2_O 5 mg/L; ZnSO_4_**·**7H_2_O 1.4 mg/L; CoCl_2_**·**6H_2_O 2 mg/L; pH 5.5) [18] with 2% glucose was used to assess the effect of DMF on hyphal growth. The effects of DMF on cellulase activity, protein concentrations, and gene expression levels were determined by medium replacement experiments performed as described previously [18], with the addition of 0–2% DMF or 1% DMSO.

### Analysis methods

Fungal hyphal growth assays were performed as described by Chen et al. [18]. For enzymatic activity, protein concentration, and biomass assays, culture supernatants and mycelia were subjected to testing as described by Chen et al. [18]. Fungal *p*NPCase and CMCase activities were measured according to the method described by Wang et al. [55]. Protein concentrations, biomass concentrations, and gene expression levels were measured according to the methods described by Chen et al. [18]. The SDS-PAGE analysis was performed on 12% Tris–HCl polyacrylamide gels using 10 μL of cell cultures treated after 4 days of cultivation.

### RNA isolation and RT-qPCR

Similar to previous reports [18], RT-qPCR was used to analyze the transcriptional levels of *egl1* (encoding endoglucanase I), *egl2* (encoding endoglucanase II), *cbh1* (encoding cellobiohydrolase I), *cbh2* (encoding cellobiohydrolase II), *xyr1* (encoding main factor XYR1), calmodulin (*cam*, GenBank: ACZ26150.1), calcineurin (*cna1*, GenBank: EGR49476.1), and calcineurin-responsive zinc finger transcription factor 1, (*crz1*, Trire2: 36391) using the 2^−ΔΔCt^ method. The sequences of the primers applied to analyze *egl1*, *egl2*, *cbh1*, *cbh2*, *xyr1*, *cam*, *cna1*, and *crz1* have been described elsewhere [18]. The sequences of the primers used to analyze *plc*-*e* are described in Additional file 11: Table S4. Gene expression levels were normalized to those of the *sar1* gene [56].

### Transcriptome analysis

Mycelia were harvested by filtration and centrifugation from cultures grown for 36 h on liquid MM containing 1% Avicel as the sole carbon source with 0 or 1% DMF supplementation. All of the mycelia of the wild-type (parental strain QM6a with 0% DMF supplementation, prepared in duplicate) and DMF (parental strain QM6a with 1% DMF supplementation, prepared in duplicate) were pooled, resulting in four samples, which were sent to a megagenomics company (Beijing, China) for library preparation and sequencing. The sequence reads were mapped to the *T. reesei* reference genome (http://genome.jgi.doe.gov/Trire2/Trire2.home.html) for bioinformatic analysis. The raw whole transcriptome shotgun sequencing data and the related protocols are available at the NCBI SRA web site (https://www.ncbi.nlm.nih.gov/sra/PRJNA510366) under accession number PRJNA510366.

### Chemical treatments

EGTA (a putative extracellular Ca^2+^ chelator) and LaCl_3_ (a plasma membrane Ca^2+^ channel blocker) were purchased from Aladdin (Shanghai, China). A final concentration of 5 mM was used for EGTA and LaCl_3_ in the *T. reesei* cultures.

### Free cytosolic Ca^2+^ labeling and detection

Ca^2+^ concentrations were assessed according to a previously described method [18]. For the fluorescence assay, the mycelia were stained with Flu-3AM (50 μM final concentration) (Sigma) at 37 °C for 30 min to visualize the amount of cytoplasmic Ca^2+^ in *T. reesei*. Then, the mycelial were washed three times with phosphate-buffered saline (PBS) to remove excess fluorophore. The images of Fluo-3 AM-labeled cells were viewed using an S Plan Fluor ELWD 20× with a 0.5 numerical aperture objective and digital sight camera on an Eclipse Ti inverted microscope system (Ti-E, Nikon, Japan) and a FITC filter (420–490 nm band-pass excitation filter and 535-nm emission filter). Average fluorescence intensity was quantified using NIS-Elements F package software.

Intracellular Ca^2+^ concentrations were measured by inductively coupled plasma mass spectrometry (ICP-MS), as described for *Ganoderma lucidum* by Xu [17] and Chen [18]. Five milliliter of culture liquid were collected at 48 h after transfer to a fresh medium with 0 or 1% DMF and subjected to filtration. Mycelia were washed with distilled water to remove any nonspecifically bound Ca^2+^ and then digested with 1 mL 68% HNO_3_. The mixture was collected via centrifugation at 12,000×*g* for 5 min. The supernatant was then filtered through a 0.22-μm membrane and diluted with 1% HNO_3_ to measure intracellular Ca^2+^ concentration. The final intracellular Ca^2+^ concentration was shown as μmol/g biomass.

### Cell permeability measurement

Cell permeability was measured according to the methods reported in Yan et al. [37], with some modifications. In brief, a 2, 3, 4-day culture of *T. reesei* in liquid MM containing 1% Avicel as the sole carbon source with the addition of 0–2% DMF or 1% DMSO were collected for subsequent experiments. The mycelia were filtered and then washed in distilled water at least three times to remove any extracellular substances. Each treatment group was divided into two, and in one group the electric conductivity of samples was measured at 0 and 3 h (*C*_0 h_ and *C*_0 h_, respectively) and in the other samples were heated in a boiling water bath for 30 min to completely destroy the cells (*C*_die_). Electrical conductivity of samples in both groups was measured using a conductivity meter and was calculated as the ratio of conductivities $\left(\left( {\frac{{C_{{3\;{\text{h}}}} - C_{{0\;{\text{h}}}} }}{{C_{\text{die}} }}} \right) \times 100\%\right)$ of both groups. The changes in the ratio of conductivities indicated changes of cell permeability.

### Statistical analysis

All experimental data were obtained from at least three independent samples with identical or similar results. The error bars indicate standard deviations (SD) from the mean of triplicates. Student’s *t* test was used to compare two samples. Duncan’s multiple-range test was used for multiple comparisons. Within each set of experiments, *p* < 0.05 was considered a significant difference.

## Additional files

**Additional file 1: Figure S1.** Effects of the addition of DMSO on cellulase production in *T. reesei* Rut-C30. a, b The effects of DMSO on CMCase activity (a) and *p*NPCase activity (b) of *T. reesei* Rut-C30. Blue bar, no DMSO added; orange bar, 1% (v/v) DMSO was added to the medium; red bar, 1% (v/v) DMF was added to the medium. Values are the mean ± SD of the results from three independent experiments. Asterisks indicate significant differences (*p < 0.05, Student’s *t* test).

**Additional file 2: Figure S2.** Microscopic assessment of the effects of DMF on the hyphal growth of *T. reesei* QM6a. The hyphae cultured in liquid Mandels’ medium were collected for microscopic assessment to detect the state of cells. The bars are 10 μm. 0% DMF, no DMF was added to the medium; 1% DMF, 1% DMF was added to the medium.

**Additional file 3: Figure S3.** Effects of DMF on the transcriptional levels of cellulase-encoding genes in *T. reesei* QM6a. a–d. Effect of DMF on the transcriptional levels of *cbh1* (a), *cbh2* (b), *egl1* (c), and *egl2* (d). Blue bar, no DMF was added to the medium; red bar, 1% (v/v) DMF was added to the medium. Values are the mean ± SD of the results from three independent experiments. Asterisks indicate significant differences from untreated strains (*p < 0.05, Student’s *t* test).

**Additional file 4: Table S1.** Sequencing statistics for whole transcriptome shotgun sequencing results from this study.

**Additional file 5: Figure S4.** Biological replicates used for the whole transcriptome shotgun sequencing analysis. Graphs representing the Pearson correlation between biological replicates of each sample. A high Pearson correlation was obtained demonstrating the reliability of whole transcriptome shotgun sequencing analysis (*r*^2^ ≥ 0.78).

**Additional file 6: Figure S5.** Volcano plot and GO enrichment analysis of up- and down-regulated genes of strains treated with 0% or 1% DMF. a. Volcano plot for differences in genes expression with 0% or 1% DMF. Red dots indicate significantly upregulated genes; green dots indicate significantly down-regulated genes; gray dots indicate non-significantly different gene expression. b. The enriched GO terms indicate biological processes, cellular components, and molecular functions in *T. reesei*. The y axis represents the enriched GO terms, and the x axis represents the number of differentially expressed genes in the term. Control: parental strain QM6a with 0% DMF supplementation; Sample 2: parental strain QM6a with 1% DMF supplementation.

**Additional file 7: Table S2.** Genes that are significantly up- or down-regulated in *T. reesei* QM6a with 1% DMF supplementation when compared with no DMF supplementation.

**Additional file 8: Table S3.** Whole transcriptome shotgun sequencing data and RT-qPCR verification of *plc*-*e* gene expression with 0 or 1% DMF added to cultures.

**Additional file 9.** Construction of Δ*plc*-*e* strains and effect of PLC-E on DMF-induced cellulase overexpression.

**Additional file 10: Figure S6.** Effects of DMF analogs on cellulase production in *T. reesei* QM6a. The effects of DMF analogs on *p*NPCase/biomass activity of *T. reesei* QM6a. Blue bar, no treatment; red bar, 1% (v/v) DMF was added to the medium; purple bar, 1% (v/v) *N*-methylformamide was added to the medium; orange bar, 1% (v/v) DMSO was added to the medium; green bar, 1% v/v *N*,*N*-dimethylamine was added to the medium; pink bar, 1% v/v *N*,*N*-dimethylacetamide was added to the medium. Values are the mean ± SD of the results from three independent experiments. Asterisks indicate significant differences from the CK (*p < 0.05, Student’s *t* test).

**Additional file 11: Table S4.** Primers used in this study.
