# Supplementary figures and images for: N,N-dimethylformamide induces cellulase production in the filamentous fungus Trichoderma reesei
Source: Biotechnol Biofuels. 2019 Feb 19;12:36. doi: 10.1186/s13068-019-1375-1 (PMC6380019; doi:10.1186/s13068-019-1375-1)

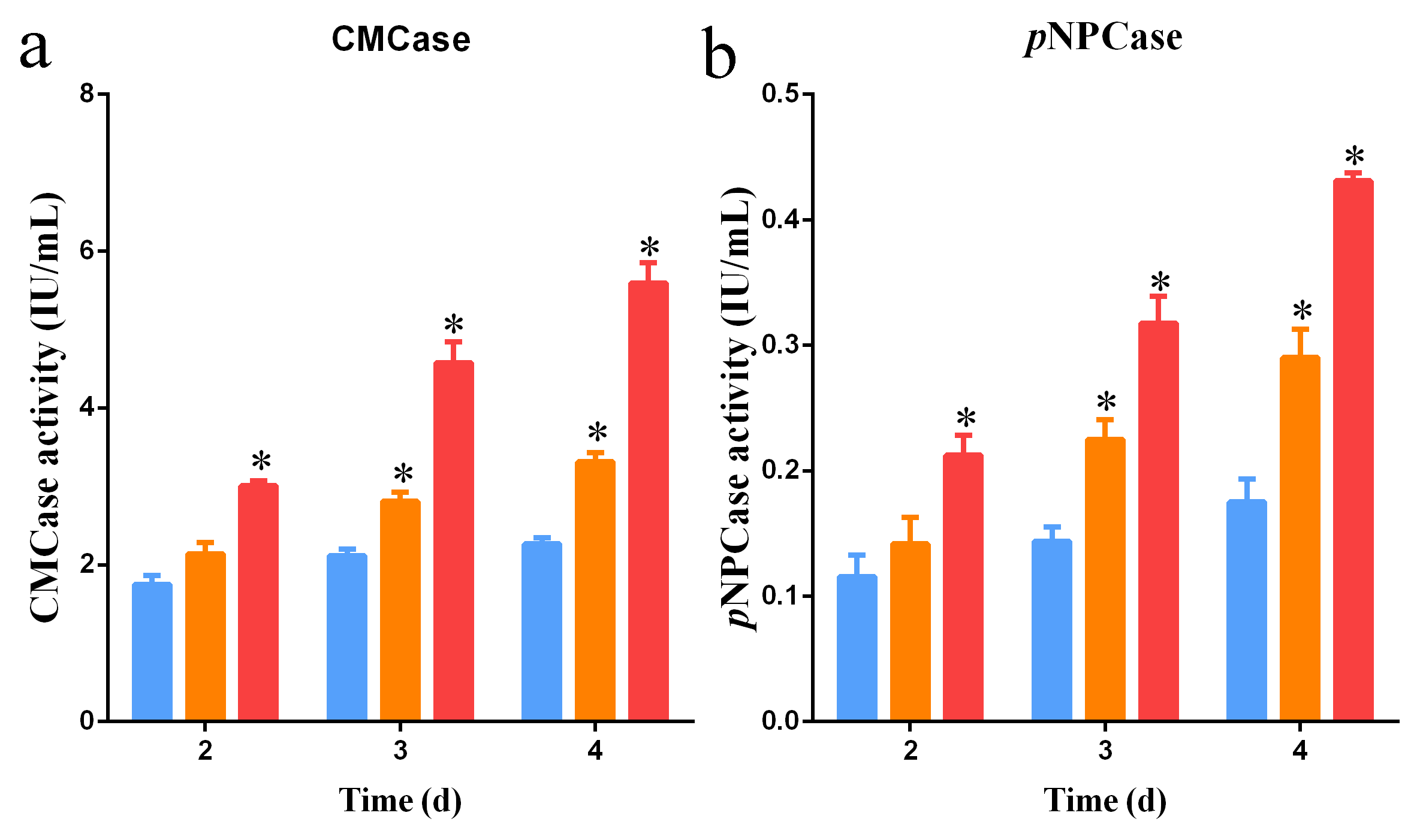

Supplement: Supplementary file 1 — Additional file 1: Figure S1. Effects of the addition of DMSO on cellulase production in T. reesei Rut-C30. a, b The effects of DMSO on CMCase activity (a) and pNPCase activity (b) of T. reesei Rut-C30. Blue bar, no DMSO added; orange bar, 1% (v/v) DMSO was added to the medium; red bar, 1% (v/v) DMF was added to the medium. Values are the mean ± SD of the results from three independent experiments. Asterisks indicate significant differences (*p < 0.05, Student’s t test). [file 13068_2019_1375_MOESM1_ESM.tif]

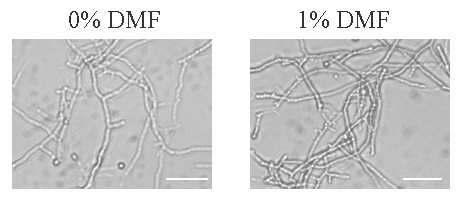

Supplement: Supplementary file 2 — Additional file 2: Figure S2. Microscopic assessment of the effects of DMF on the hyphal growth of T. reesei QM6a. The hyphae cultured in liquid Mandels’ medium were collected for microscopic assessment to detect the state of cells. The bars are 10 μm. 0% DMF, no DMF was added to the medium; 1% DMF, 1% DMF was added to the medium. [file 13068_2019_1375_MOESM2_ESM.tif]

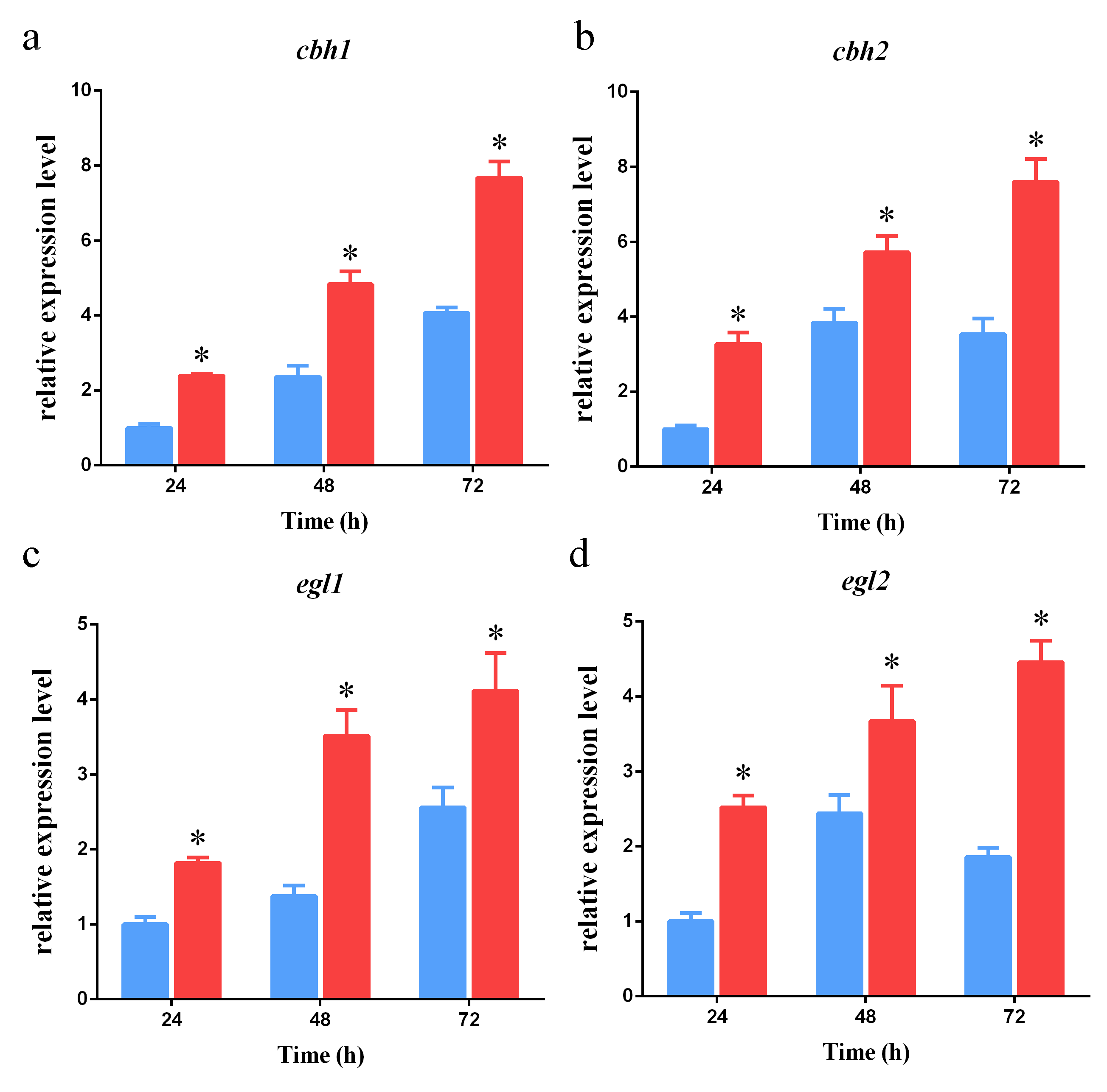

Supplement: Supplementary file 3 — Additional file 3: Figure S3. Effects of DMF on the transcriptional levels of cellulase-encoding genes in T. reesei QM6a. a–d. Effect of DMF on the transcriptional levels of cbh1 (a), cbh2 (b), egl1 (c), and egl2 (d). Blue bar, no DMF was added to the medium; red bar, 1% (v/v) DMF was added to the medium. Values are the mean ± SD of the results from three independent experiments. Asterisks indicate significant differences from untreated strains (*p < 0.05, Student’s t test). [file 13068_2019_1375_MOESM3_ESM.tif]

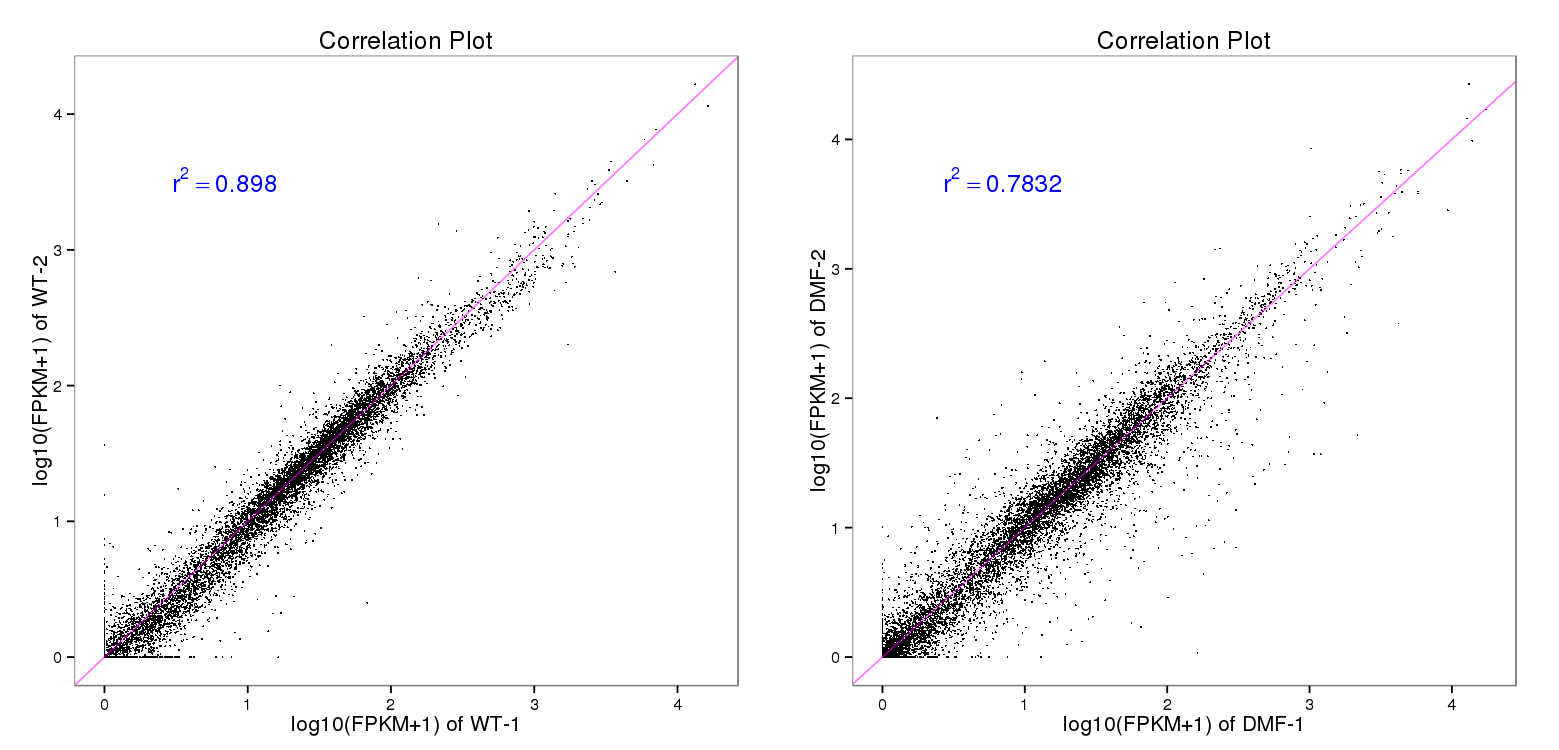

Supplement: Supplementary file 5 — Additional file 5: Figure S4. Biological replicates used for the whole transcriptome shotgun sequencing analysis. Graphs representing the Pearson correlation between biological replicates of each sample. A high Pearson correlation was obtained demonstrating the reliability of whole transcriptome shotgun sequencing analysis (r2 ≥ 0.78). [file 13068_2019_1375_MOESM5_ESM.tif]

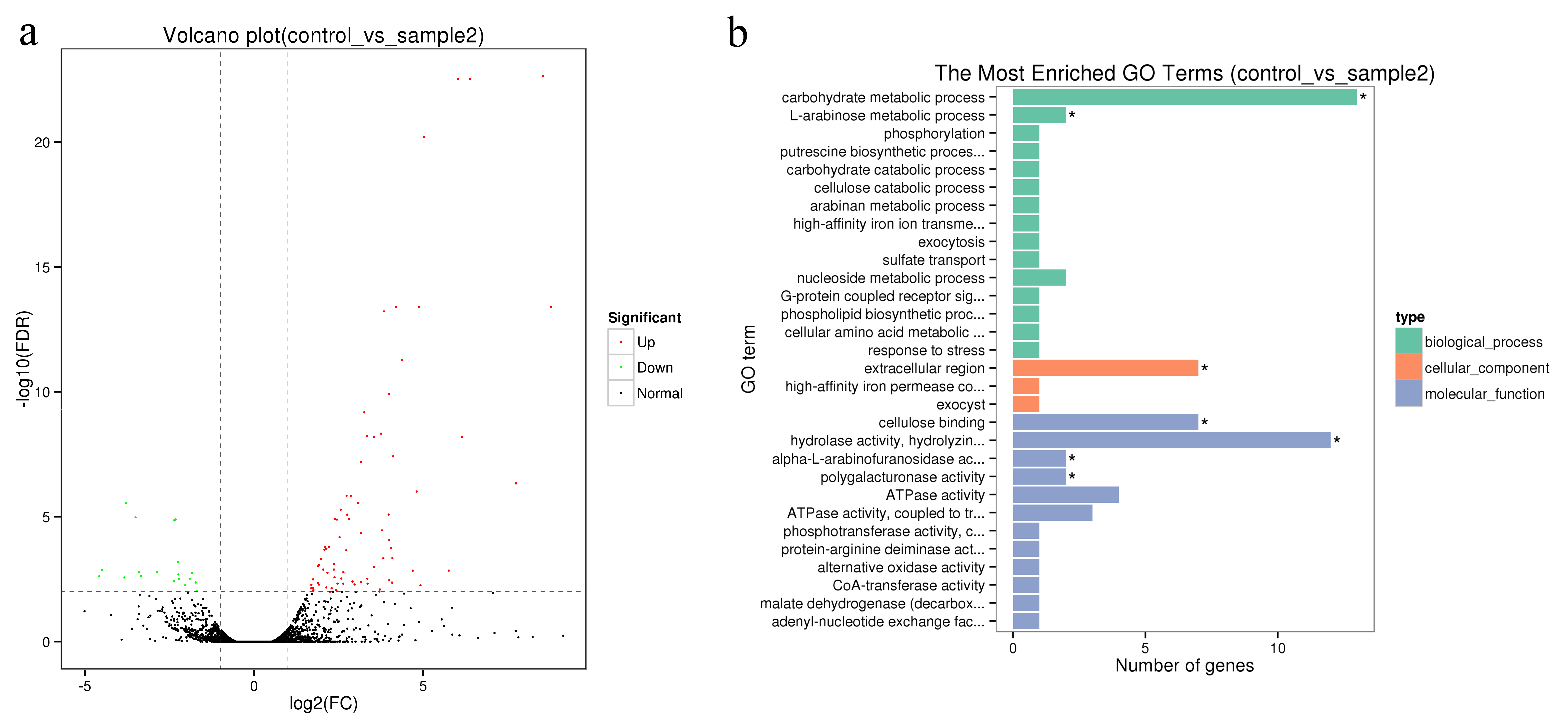

Supplement: Supplementary file 6 — Additional file 6: Figure S5. Volcano plot and GO enrichment analysis of up- and down-regulated genes of strains treated with 0% or 1% DMF. a. Volcano plot for differences in genes expression with 0% or 1% DMF. Red dots indicate significantly upregulated genes; green dots indicate significantly down-regulated genes; gray dots indicate non-significantly different gene expression. b. The enriched GO terms indicate biological processes, cellular components, and molecular functions in T. reesei. The y axis represents the enriched GO terms, and the x axis represents the number of differentially expressed genes in the term. Control: parental strain QM6a with 0% DMF supplementation; Sample 2: parental strain QM6a with 1% DMF supplementation. [file 13068_2019_1375_MOESM6_ESM.tif]
