# Supplementary material for: N,N-dimethylformamide induces cellulase production in the filamentous fungus Trichoderma reesei
Source: Biotechnol Biofuels. 2019 Feb 19;12:36. doi: 10.1186/s13068-019-1375-1 (PMC6380019; doi:10.1186/s13068-019-1375-1)
Supplement: Supplementary file 4 — Additional file 4: Table S1. Sequencing statistics for whole transcriptome shotgun sequencing results from this study. [file 13068_2019_1375_MOESM4_ESM.docx]

**Table S1**. Sequencing statistics for whole transcriptome shotgun sequencing results from this study.

| Sample | Raw Reads | Clean Reads | Raw Bases(Gb) | Clean Bases(Gb) | Effective Rate (%) | Mapped Reads |
| --- | --- | --- | --- | --- | --- | --- |
| WT-1 | 46006536 | 44342540 | 6.90 | 6.65 | 96.38 | 41,623,363(93.87%) |
| WT-2 | 50019484 | 49087666 | 7.50 | 7.36 | 98.14 | 46,316,299(94.35%) |
| DMF-1 | 47644208 | 46759924 | 7.15 | 7.01 | 98.14 | 44,330,453(94.80%) |
| DMF-2 | 50345166 | 49423624 | 7.55 | 7.41 | 98.17 | 46,928,934(94.95%) |

WT: parental strain QM6a with 0% DMF supplementation performed in triplicate (indicated by −1 and −2).

DMF: parental strain QM6a with 1% DMF supplementation performed in triplicate (indicated by −1 and −2).
