# Supplementary material for: N,N-dimethylformamide induces cellulase production in the filamentous fungus Trichoderma reesei
Source: Biotechnol Biofuels. 2019 Feb 19;12:36. doi: 10.1186/s13068-019-1375-1 (PMC6380019; doi:10.1186/s13068-019-1375-1)
Supplement: Supplementary file 8 — Additional file 8: Table S3. Whole transcriptome shotgun sequencing data and RT-qPCR verification of plc-e gene expression with 0 or 1% DMF added to cultures. [file 13068_2019_1375_MOESM8_ESM.docx]

**Table S3. Whole transcriptome shotgun sequencing data and qRT-PCR verification of the transcription levels of the *plc* gene with DMF added.**

| **Gene ID** | **Gene name** | **Gene product** | **log_2_ fold change (DMF^a^/** **WT^b^)** | **log_2_ fold change (DMF/WT)** |
| --- | --- | --- | --- | --- |
|  |  |  | **RNA-seq^c^** | **qRT-PCR^d^** |
| Trire2:21960 | *plc-e* | phospholipase C | 2.11 | 2.8 |

^a^ DMF, gene expression level in parental strain QM6a with 1% DMF supplementation.

^b^ WT, gene expression level in parental strain QM6a with 0% DMF supplementation.

^c^ Results from FPKM values shown in the RNA-Seq data.

^d^ Results of relative expression levels obtained by qRT-PCR assays. The values are means of the results from three independent experiments.
