# Supplementary material for: N,N-dimethylformamide induces cellulase production in the filamentous fungus Trichoderma reesei
Source: Biotechnol Biofuels. 2019 Feb 19;12:36. doi: 10.1186/s13068-019-1375-1 (PMC6380019; doi:10.1186/s13068-019-1375-1)
Supplement: Supplementary file 9 — Additional file 9. Construction of Δplc-e strains and effect of PLC-E on DMF-induced cellulase overexpression. [file 13068_2019_1375_MOESM9_ESM.docx]

**1. Construction of plasmids and Δ*plc-e* strains**

To construct a *plc-e* deletion mutant, the 791-bp upstream and 745-bp downstream regions of *plc-e* were generated from the genome of *T. reesei* QM6a using KOD-Plus-Neo (TOYOBO, Japan). First, the upstream fragment was ligated into the *Pac*I and *Xba*I linearized LML2.0 [1] using the ClonExpressTM II One Step Cloning Kit (Vazyme, Nanjing, China) to form pF*plc-e*. Subsequently, the downstream fragment was inserted into *Swa*I-linearized pF*plc-e* to form the binary vector pD*plc-e* for the knockout of *plc-e* in QM6a using *Agrobacterium*-mediated transformation [2]. Strains were selected using hygromycin B and cefotaxime on Mandels’ medium. Then the marker was excised using the method described by Zhang [61]. The putative *plc-e* disruption mutants (Δ*plc-e*) generated by double crossover were verified by diagnostic PCR using the primers plc-e-CF and plc-e -CR and plc-e -OF and plc-e -OR. The sequences of the primers used are described in Table S1.

| **Table S1 Primers used in this study.**   \| Primer \| oligos Sequences (5’ to 3’) \| \| --- \| --- \|   **Construction of *Trplc-e* gene deletion vector** | |
| --- | --- | --- | --- |
| plc-e-D1 | ATTACGAATTCTTAATTAATGTCTCAGTCTCCACACCTCTC |
| plc-e-D2 | CATTATACGAAGTTATTCTAGAGCAGCCAAGAAGCAGGATAGC |
| plc-e-D3 | ACTAGTGAGCTCATTTATTGACATGCAGAAGGCGTGAT |
| plc-e-D4 | AGTGCCAAGCTTATTTCTAGAAGCGAGTGACTGATGGC |
| **Verification of the *Trplc-e* gene deletion mutants** | |
| plc-e-CF | AGTAGTGTTCGGGAGGCTTACG |
| plc-e-CR | AGATGATTCTTTACTGGTGCCGTG |
| plc-e-OF | GACCACAACTCGCAGATTCTCT |
| plc-e-OR | GCTCACGCTATACACCTTCCAG |

**2. Effect of PLC-E on DMF-induced cellulase overexpression.** *p*NPCase/biomass activity of *T. reesei* QM6a and Δ*plc-e* strains supplemented with 0% or 1% DMF. Red bar, adding 1% (v/v) DMF in *T. reesei* QM6a; blue bar, adding 0% (v/v) DMF in *T. reesei* QM6a; purple bar, adding 1% (v/v) DMF in Δ*plc-e*; green bar, adding 0% (v/v) DMF in Δ*plc-e*. Values are the means ± SD of the results from three independent experiments. Asterisks indicate significant differences (**p* < 0.05, Student’s *t* test).

**

**

**References**

1. Zhang L, Zhao X, Zhang G, Zhang J, Wang X, Zhang S, Wang W, Wei D. Light-inducible genetic engineering and control of non-homologous end-joining in industrial eukaryotic microorganisms: LML 3.0 and OFN 1.0. Sci Rep. 2016;6:20761.

2. Zhang G, Liu P, Wei W, Wang X, Wei D, Wang W. A light-switchable bidirectional expression system in filamentous fungus *Trichoderma reesei*. J Biotechnol. 2016;240:85-93.
