# Supplementary material for: N,N-dimethylformamide induces cellulase production in the filamentous fungus Trichoderma reesei
Source: Biotechnol Biofuels. 2019 Feb 19;12:36. doi: 10.1186/s13068-019-1375-1 (PMC6380019; doi:10.1186/s13068-019-1375-1)
Supplement: Supplementary file 11 — Additional file 11: Table S4. Primers used in this study. [file 13068_2019_1375_MOESM11_ESM.docx]

**Table S4 Primers used in this study.**

| Primer | oligos Sequences (5’ to 3’) |
| --- | --- |
| **Quantitative RT-PCR analysis** | |
| Qplc-1 | CGGTGGGCGAGCACTACGAA |
| Qplc-2 | TCGGCGTCCTGGAACTGCTG |
